# A life dedicated to research and ideal: Johannes Müller between empirical universality and idealistic vitalism mirrored in lecture notes from 1851

**DOI:** 10.1007/s12064-024-00422-7

**Published:** 2024-08-19

**Authors:** Ulrich Zeller, Ingmar Werneburg

**Affiliations:** 1 Lebenswissenschaftliche Fakultät, Albrecht Daniel Thaer-Institut für Agrar- und Gartenbauwissenschaften, Humboldt-Universität zu Berlin, Luisenstraße 53, 10117 Berlin, Germany; 2 Paläontologische Sammlung, Fachbereich Geowissenschaften, Eberhard Karls Universität, Hölderlinstraße 12, 72074 Tübingen, Germany; 3 Senckenberg Center for Human Evolution and Palaeoenvironment, Eberhard Karls Universität, Sigwartstraße 10, 72076 Tübingen, Germany

**Keywords:** Transcription, Pre-Darwinian biology, Zoology, Comparative anatomy, Empiricism

## Abstract

Until the mid-nineteenth century, "physiology" was a comprehensive theory of life, expounded and shaped by Johannes P. Müller (1801–1858). Biologists and medical doctors still refer to him today. In the summer term of 1851, Müller gave a lecture on the Comparative Anatomy of animals. This lecture was attended and recorded by Ernst Zeller (1830–1902), a future physician and zoologist, and has recently been published together with a German transcript. In this paper, we situate Johannes Müller within the intellectual history of his time. Through his "empirical idealism," we show how he opposed the speculative tendencies of the romantic understanding of nature, the emerging evolutionism, and the growing splits in the natural sciences. Müller focused on recognizing living nature as a whole and realizing ideal "phenomena" through his empirical research. He considered the notion of the soul of the world. Müller's lecture transcript serves as a poignant testament to German scientific culture in the mid-nineteenth century, a few years before the publication of Darwin's Origin of Species. It also provides valuable insights into the self-contained epistemological foundations of morphology.

## Introduction

### Johannes Müller and the value of lecture notes for the history of science

**Fig. 1:**
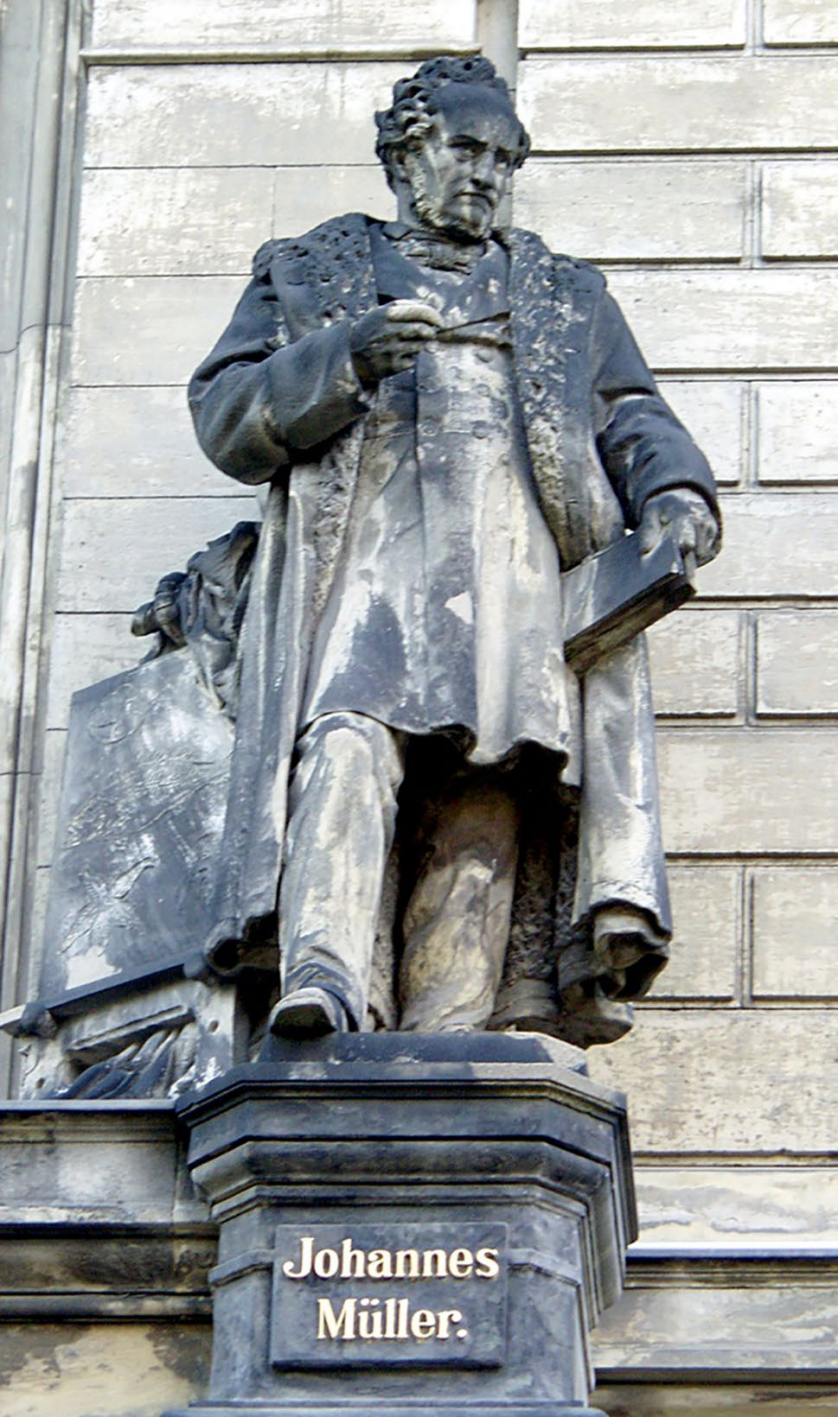
Statue of Johannes Müller (1801–1858) above the entrance to the Museum für Naturkunde in Berlin in 2007. © Raimond Spekking/CC BY-SA 4.0 (via Wikimedia Commons)

Johannes Peter Müller (1801–1858; Fig. 1) is considered one of the most important natural scientists of the nineteenth century and is generally regarded as the father of modern biology and medicine. He first served as a "professor of physiology" in Bonn, Germany, and then continued his tenure at the University of Berlin, Germany, from 1833 to 1858 (Table 1). Müller is widely considered to be the last scholar of his time to comprehensively explore the fields of anatomy, functional physiology, and pathology, i.e., the whole of theoretical medicine, in research and teaching (Meyer-Abich 1949). After his death, his students founded several scientific disciplines, and Müller was the last to unite classical universality in one person (Otis 2007).

**Table 1:** Scientific biography of Johannes Müller

Year	Event
1801	Born July 14th in Koblenz, a student of the natural philosopher and Catholic journalist Joseph Görres (1776–1848) at the local high school
1818	Served with the pioneers in Koblenz
1819–23	Studied medicine at the University of Bonn, founded in 1818, where he was a student of the anatomist and physiologist August Franz Josef Karl Mayer (1787–1865)
1822	Doctorate in Bonn on laws of motion in the animal kingdom (“De phoronomia animalium “) and moved to the University of Berlin, founded in 1809, where he attended lectures by the anatomist Karl Asmund Rudolphi (1771–1832) in 1823–24
1823	The first award-winning scientific work on the physiology (especially breathing) of the fetus, written as a student, appears
1824	Habilitation in Bonn in physiology and Comparative Anatomy with the inaugural lecture “On the need of physiology for a philosophical view of nature” on October 19 1824; lecturer until 1827; becomes a member of the Leopoldina; October 19th: first encounter with Goethe (enthusiasm)
1826	Appointed Associate Professor in Bonn; “ On the fantastic visual phenomena” and “On the comparative physiology of the visual sense” appear, in which the “Law of the specific sensory energies” arises
1828	Second and final encounter with Goethe (disillusionment)
1830	Appointed full professor in Bonn; the “History of the Formation of the Genitalia” with the description of the Müllerian duct and “De glandularum secernentium structura penitiori” as well as the habilitation thesis “De Ovo humano atque Embryos Observations anatomicae “
1832	First microscopic examinations; the “Contributions to the anatomy and natural history of amphibians” appear
1833	Call to Freiburg rejected
1833ff	The two-volume main work, the “Handbook of Physiology,” appears in several parts
1833	Becomes Rudolphi's successor at the University of Berlin; The “Comparative Anatomy of Myxinoids” was published from 1834 to 1843
1838	“On the finer structure and forms of pathological tumors” appears
1839	“On the compensation of physical forces on the human vocal organ” appears
1840	With Franz Hermann Troschel he publishes the work “On the genera of the ophiurs”
1841	With Friedrich Gustav Jakob Henle he publishes the “Systematic Description of Plagiostomes”
1842	With Franz Hermann Troschel he published the “System of the Asterides”
1845–1849	With Franz Hermann Troschel he publishes the “ Horae” in two parts ichthyological: description and illustration of new fish”
1847	Honorary member (Honorary Fellow) in the Royal Society of Edinburgh
1849	Elected to the American Academy of Arts and Sciences
1852	“About Synapta digitata and on the emergence of snails in Holothuria” appears
1853	Receives the Bavarian Order of Maximilian for Science and Art; “On the general plan in the development of echinoderms” appears
1854	Received the Copley Medal from the Royal Society in London and the Prix Cuvier from the Paris Academy
1853	Summary of the development of the echinoderms: “About the general plan in the development of the echinoderms” read in the Königl. Academy of Sciences in Berlin
1858	† April 28th in Berlin

Johannes Müller was characterized by a highly empirical and rational research agenda, which set him apart from several contemporaries of Romantic *Naturphilosophie* in Germany. In his scientific books and articles, Müller was relatively silent about his conceptual and philosophical approaches to science (Koller 1958; Otis 2007). This is noteworthy because he was embedded in the Catholic faith and can be considered a vitalist. Müller died one year before the publication of Darwin's (1859) “On the Origin of Species,” but he was already aware of evolutionary ideas inspired by the Cuvier-Geoffroy debate of 1830. Yet little is known about the epistemological approach that inspired Müller and how he navigated the traditions and trends of his time.

In the summer semester of 1851, Johannes Müller gave a lecture on 'Comparative Anatomy [of Animals]' in Berlin, which was attended by Ernst Zeller (1830–1902, Fig. 2), a future physician and zoologist, who also recorded the lecture for his student purposes. The lecture notes belonged to the personal inheritance of the first author (Ulrich Zeller) and have now been given to the Berlin University Library and bear the inventory number 8858 (Figs. 3, 4, 5). A digital copy of the notes is available there at the following address: https://nbn-resolving.org/urn:nbn:de:kobv:11-717937. A facsimile, together with a German transcription, has recently been published (Zeller and Werneburg 2024a, b).

**Fig. 2:**
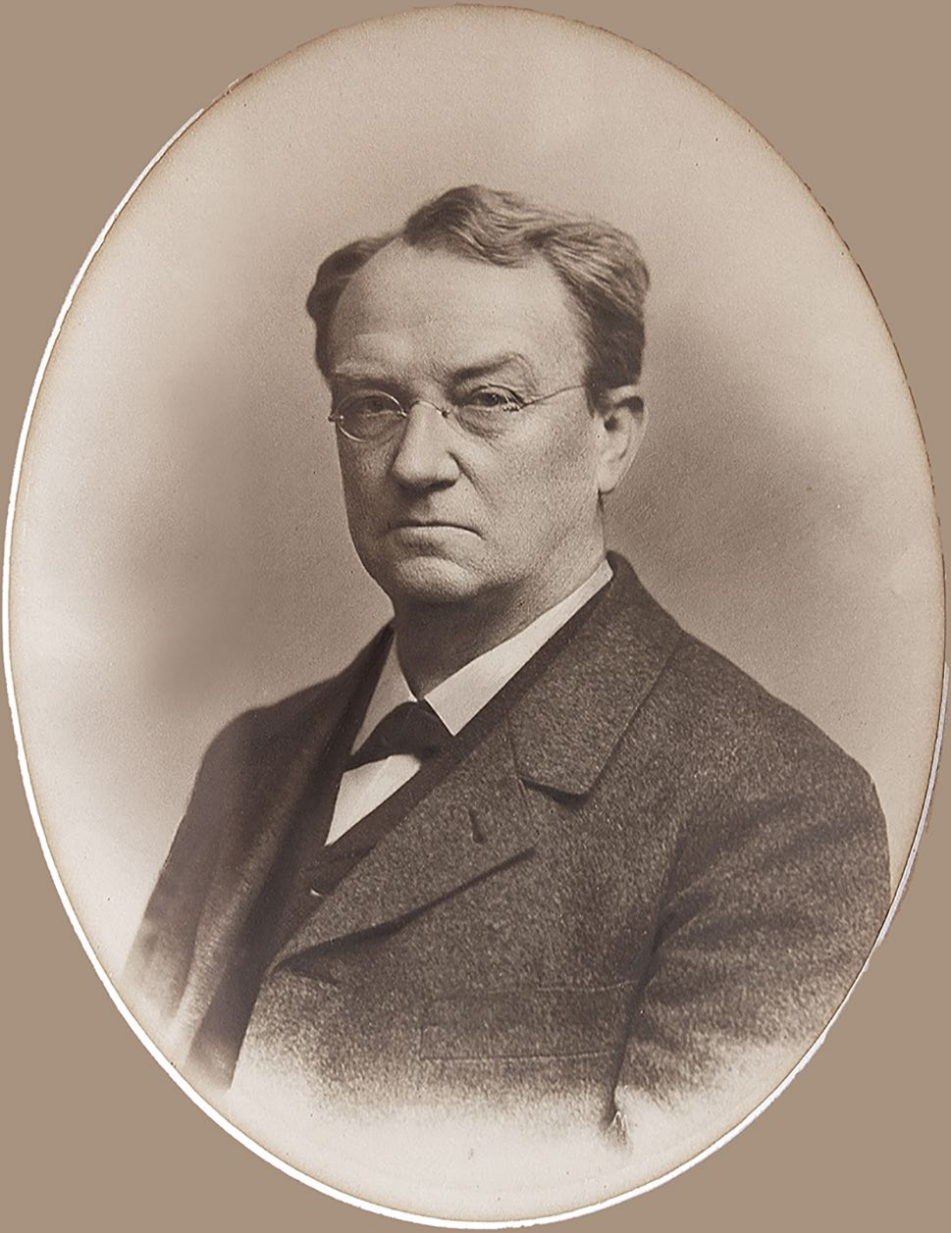
Ernst von Zeller (1830–1902), no year. Private archive of Ulrich Zeller. See also: Klunzinger (1903)

**Fig. 3:**
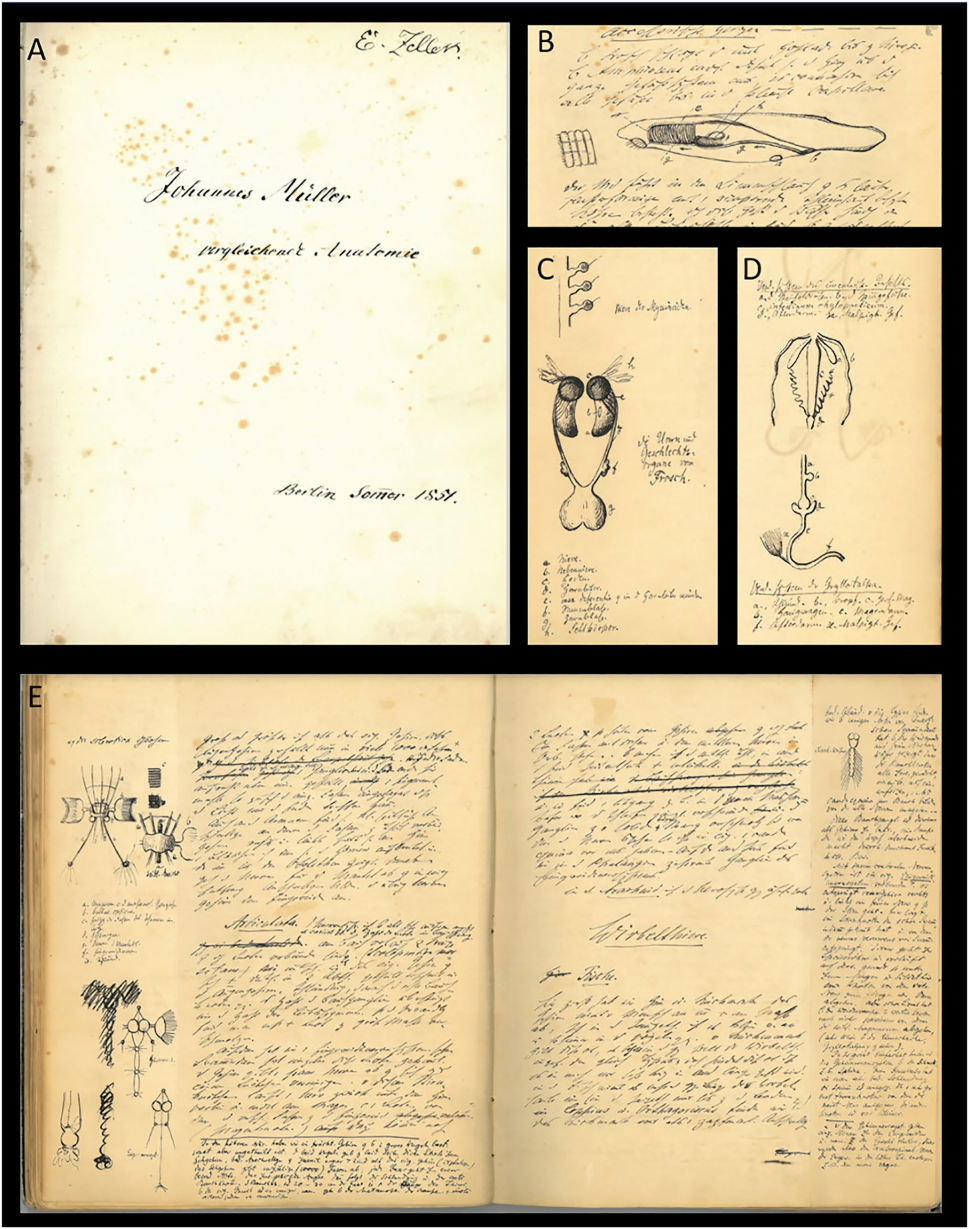
The lecture notes 1/3. (**A**) full Folio 1r, showing the title page of the script; (**B**) Folio 26v, showing the blood vessel system of the lancet fish; (**C**) Folio 54v, with “Niere der Myxinoiden” (“kidney of myxinoids”) and “Die Harn- und Geschlechtsorgane vom Frosch” (“the excretory and sexual organs of the frog”); (**D**) Folio 52r, with top: “Verd‹auungs›system des unentwik‹elten› Insekts.” (“digestion system of the undeveloped insect”) and below: “Verd‹auungs›system der *Gryllotalpa*.” (digestive system of *Gryllotalpa*); E) Folios 32v and 33r from the section “nervous system”

**Fig. 4:**
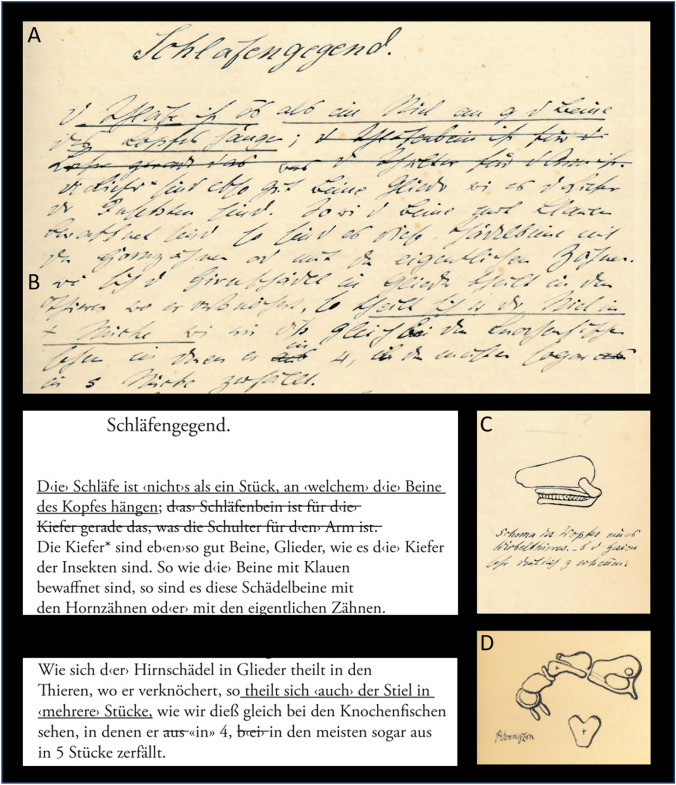
The lecture notes 2/3. (**A**–**C**) Folio 8r; (**D**) Folio 10v. Translations: (**A**) temporal skull region. The temporal region is nothing than a part, at which the legs of the head hang. The jaws* [of vertebrates] are basically legs, extremities, such as the jaws are in insects. Just as the jaws are wapponed with claws, the temporal bone has horny teeth or the true teeth. (**B**) As the neurocranium is separated in parts in the animals, where it ossifies, its stem also separates into several parts, as we can see it in bony fishes in which it of 4, or in most [cases] even of 5 parts

**Fig. 5:**
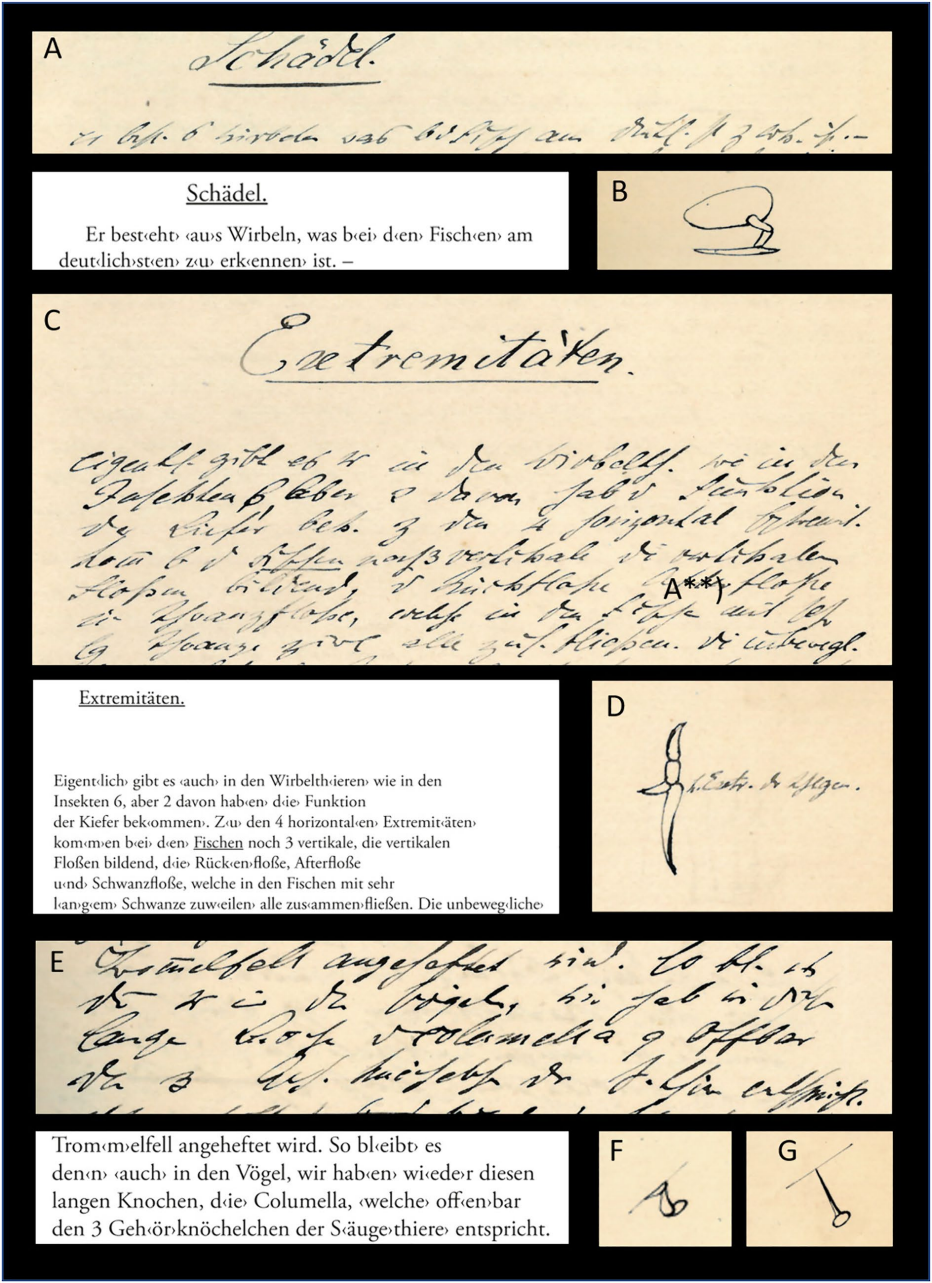
The lecture notes 3/3. (**A**) Folio 6v; (**B**) Folio 8v; (**C**) Folio 17v; (**D**) Folio 18r („h < intere > Extr < emität > der Schl < angen > “ = hind limb of snakes); (**E**–**G**) Folio 40r, with F showing three middle ear ossicles of mammals and G showing the columella (and extracolumella) of reptiles. Translations: (**A**) Skull. It consists of vertebrae, which is best visible in fish. (B) Extremities. Actually, there are 6 [extremities] in vertebrates—as in insects—but 2 of them take the function of the jaws. In addition to the 4 horizontal extremities, there are 3 additional vertical ones in fish forming the vertical fins, back fin, after fin, and tail fin, which may partly fuse in fishes with very long tails. The unmovable […]. E) […] the tympanic membrane is fixed. In such shape, it remains in birds: we can find, again, this long bone, the columella, which, apparently, equals the 3 ear ossicle of mammals

As a reliable source of scientific history, lecture notes are of particular value. Recent studies of the lecture notes of the morphologists Ernst Haeckel and Carl Gegenbaur, for example, illustrate the great advances in Comparative Anatomy in the early years after the Darwinian revolution of 1859 (Hoßfeld et al. 2022a, b; Werneburg et al. 2022a, b, c). In lectures, an instructor's speaking style differs from that of a written text, which is subject to greater scrutiny. The use of lecture notes allows for a more engaging and nuanced understanding of a researcher's thought process, which can otherwise be obscured by highly rational scientific texts, as in the case of Müller. In the end, official documents and unauthorized lecture notes together provide a more complete picture of an individual's intellectual entity. This approach provides unique and otherwise hidden access to a researcher's intrinsic motivation to pursue an individual scientific agenda.

After introducing Müller's scientific work and discussing the career of his student Ernst Zeller, we summarize the lecture notes and refer to some aspects of the scientific history of selected topics presented in the lecture. In the second part of the article, based on the lecture notes and other sources, we discuss the intellectual profile of Johannes Müller as a key figure in the history of natural science in the period just before the Darwinian revolution.

### The scientific work of Johannes Müller

Müller, along with Johann Lucas Schönlein (1793–1864), is considered the founder of the new scientific medicine in Germany (Schnabel 1934). Müller's groundbreaking research in functional and comparative anatomy, pathology, and marine biology is widely regarded as pioneering in these fields. His students, namely Rudolph Virchow (1821–1902; in pathology), Carl Gegenbaur (1826–1903; in Comparative Anatomy), and Ernst Haeckel (1934–1919; in zoology), gained worldwide recognition. Müller is undoubtedly one of the most prominent figures in medicine, not only in Germany but beyond its borders. Müller and Schönlein founded the "Berlin School" of medicine, which led to the significant era of the Charité. This era witnessed the achievements of several pioneers, including Robert Koch (1843–1910), who discovered the tuberculosis pathogen, and Emil von Behring (1854–1917) and Paul Ehrlich (1854–1915), who invented passive antitoxic therapy for diphtheria (Fischer and Ganten 2021).

**Fig. 6:**
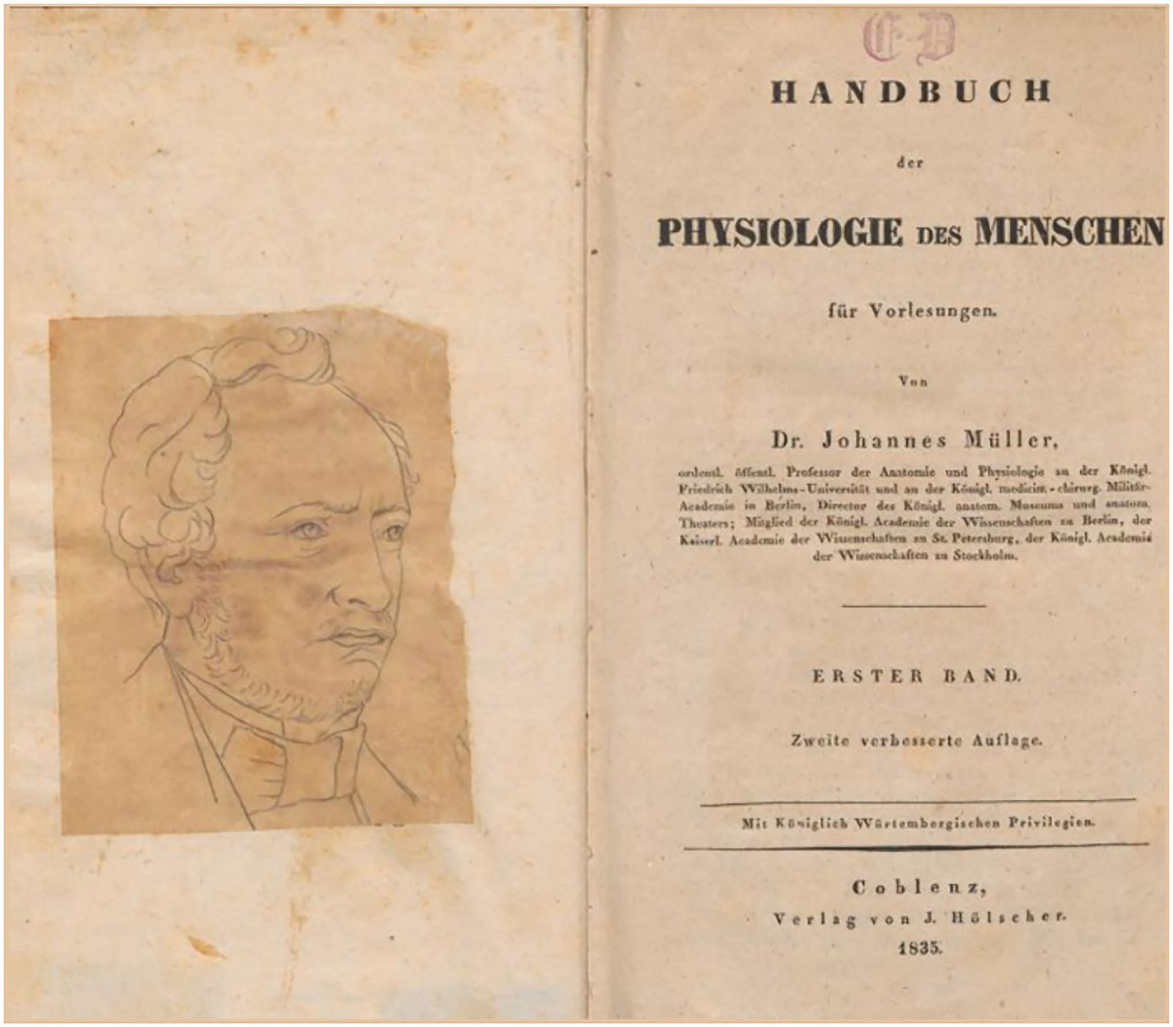
Cover image of the “Handbook of Human Physiology for Lectures” (2nd edition, Volume 1) from 1835. Ex libris Eugène Dubois (1858–1940) from 1881, with a drawing of Johannes Müller’s portrait. Scan by the Naturalis Biodiversity Center, Leiden/Netherlands, for archive.org (ark:/13960/t3422742z). The drawing is borrowed from the last portrait of Johannes Müller, a photograph by S. Friedländer in the summer of 1857 and lithographed by P. Rohrbach in 1858

Johannes Müller's most important contributions to this field of science (e.g., Carus 1872, Koller 1958, and Starck 1981) are the (1) fundamental morphological descriptions of "lower vertebrate" species, which are considered central to the understanding of vertebrate anatomy (cf. Fig. 3B; Müller 1832, 1834, 1840, 1841, 1843, 1844; Müller and Henle 1841; Müller), (2) the function and structure of sensory organs (Müller 1826a, 1828, 1831), (3) the anatomy of the vocal apparatus (Müller 1839) and sexual organs (Müller 1830), (4) paleontology (Müller 1846, 1849), (5) human physiology (Fig. 6; Müller 1833ff) and pathology (Müller 1838), and (6) the systematics (Müller 1858), anatomy (cf. Fig. 3D; Müller 1825, 1829), and life history (Müller 1848, 1852a, b, c) of several invertebrate species.

**Fig. 7:**
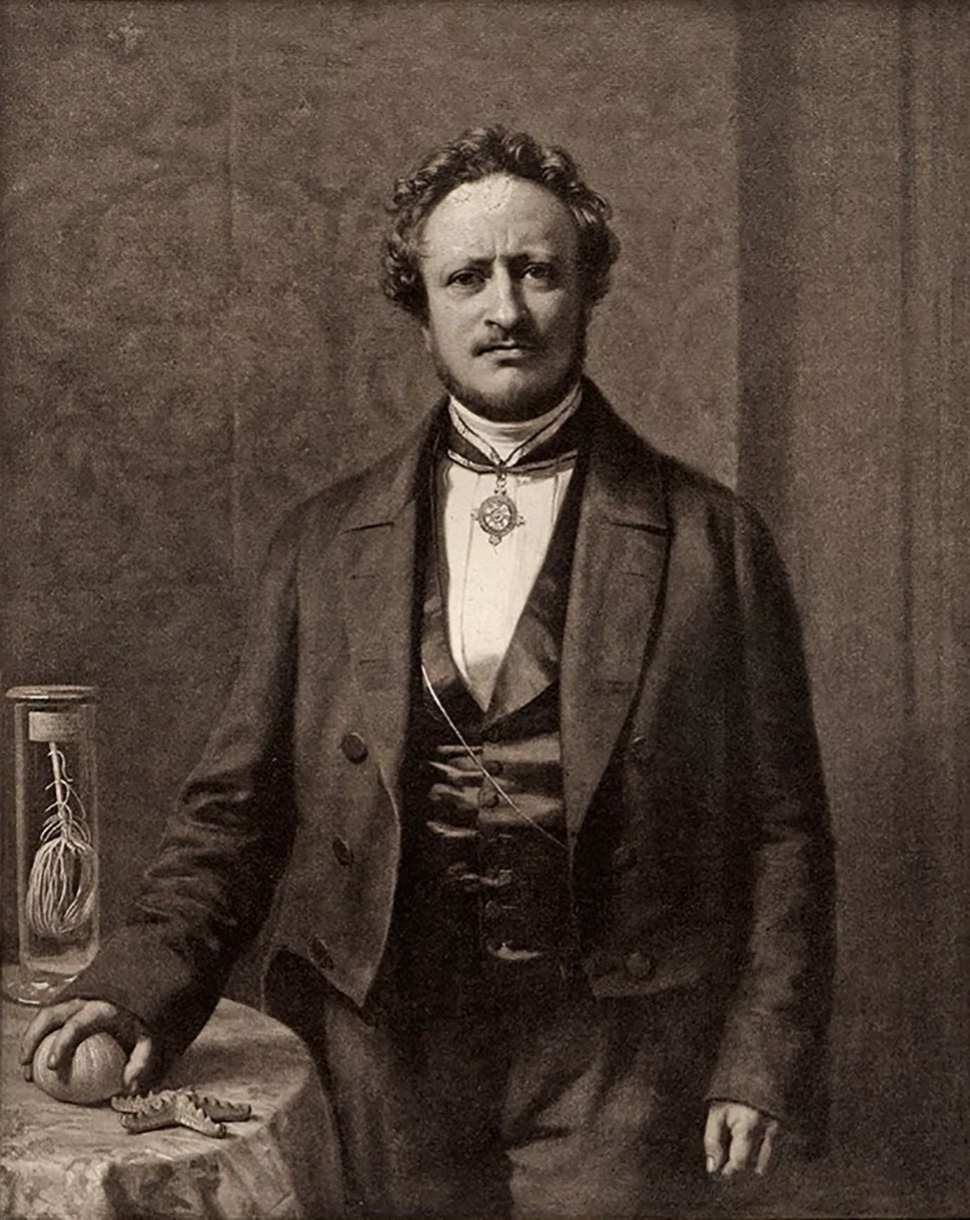
Johannes Müller, painting by Karl Begas the Younger (Photographic Society Berlin) 1856. On the table are a starfish, a sea urchin and in the glass a sea lily, all of which belong to the echinoderms that Müller had studied for years

**Fig. 8:**
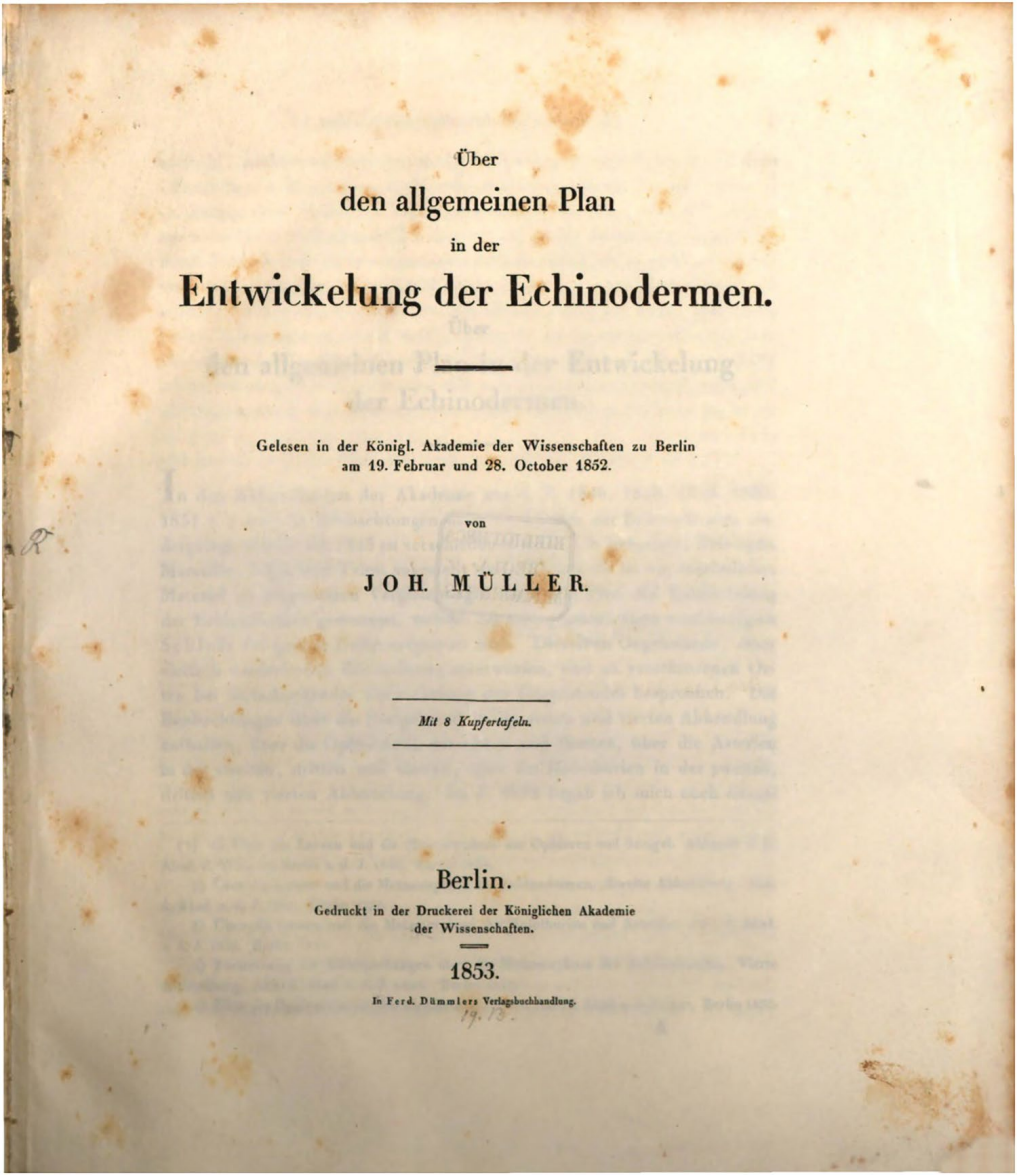
Title page “On the general plan in the development of echinoderms” (Müller 1852b [1853])

Müller's 1851 lecture on Comparative Anatomy is often taken as evidence of his transition from physiology to morphology, or from being a physiologist to being a morphologist. However, it is important to note that in the early nineteenth century, "physiology" was used more broadly, much like modern "biology" or "life sciences" (Koller 1958; Nyhart 1995). Furthermore, Müller's scientific work was from the beginning characterized by a holistic approach. Nevertheless, he considered the anatomical-morphological direction of physiology to be the most effective (Müller 1834; quoted in Meyer-Abich 1949: p. 275). It was the emergence of physiology as a reductionistic and atomistic approach, as we know it today, that prompted Müller, especially during his Berlin years, to shift from experimentation (Müller 1826b) to descriptive observation. Since 1841 he had been working on marine biology, which became more important during this innovative phase (Fig. 7; Koller 1958; Otis 2007), and his research on the life cycle of echinoderms (Müller [Bibr CR76][Bibr CR79] [1853]; Fig. 8) served as a refuge and substitute for what he perceived as a "drifting" physiology. His work made it possible to observe fundamental, essential characteristics of living things. The lecture that Ernst Zeller attended and recorded during the summer term of 1851 fits the criteria of this fruitful period of creativity.

### The Zeller family from Württemberg and Johannes Müller in Berlin

Ernst Zeller (Fig. 2) studied medicine in Tübingen, Germany, and later, at the age of 21 years, traveled to Berlin to attend a lecture by Johannes Müller in 1851. His subsequent scientific work was influenced by the impressions he gained during this time, which he continued while working as a physician. Albert Zeller (1804–1877) was the father of Ernst Zeller, and the grandfather was Chief Justice Johann Friedrich Zeller, a direct descendant of the Maulbronn prelate Johannes Zeller (1620–1694). As a physician and psychiatrist, Albert Zeller may have been influenced by his mother, Johanna Regine Andreae.

Albert Zeller found Schelling’s (1798) Romantic *Naturphilosophie*, which dominated medicine at the time, and its speculative excesses unsatisfactory. Psychiatry seemed divided between "somaticians" and "psychologists" (Ackerknecht 1977), and a comprehensive account of physiology was lacking. The breakthrough of scientifically oriented medicine in the nineteenth and twentieth centuries came with the publication of Johannes Müller's "Handbuch der Physiologie" (1833ff) and Johann Lucas Schönlein's work, which laid the foundation for the "natural history school". Meanwhile, Albert Zeller focused on sensory physiology and later developed his theory of unity psychosis (G. Zeller 1961, 2007). This can also be understood in light of Müller's pantheistic belief in a universal world soul, as well as his proposal that life and soul correspond to matter, but are not composed of individual components. Following Müller's statement that the sentient and imaginative soul functions only in organized matter, the unity of psychosis is attributed to the polar structuring of the nervous system into a somatic pole (ganglia) and a pneumatic pole (cerebrum). This leads to the transition of different stages of mental illness, described as "transmotio morborum" (Vliegen 1980). Conversely, unlike Müller, Albert Zeller was an animist, not a vitalist (G. Zeller 2007). The traces of subjective natural philosophy were still present in his views, especially on the subject of the relationship between body and soul ("the eye does not see, only the soul," G. Zeller 2007, Vol. 2: p. 34). They were only omitted by his son Ernst Zeller.

Ernst Zeller was granted what his father unfortunately missed: a personal encounter with Johannes Müller. This meeting took place during the summer semester of 1851 in Berlin, where the student was staying in the cosmopolitan home of his grandfather, the publisher and bookseller Georg Andreas Reimer (1776–1842). Ernst Zeller attended and recorded Johannes Müller's lecture on "Comparative Anatomy" in its entirety. Müller published several times with Reimer, including the monographs on tumors and zeuglodonts (Müller 1838, 1849). It is plausible to assume that there was a meeting of all those involved in the Reimer household in 1851. The considerable influence of Johannes Müller on the aspiring medical student directed Ernst Zeller toward a scientific approach to medicine. However, Zeller's rigorous rationalism was limited by psychiatry and the still unresolved mind–body problem. Here, as successor to his father, he pragmatically reformed and oversaw the reconstruction of the Winnenthal institution in Winnenden near Stuttgart, Germany, based on logical principles and for the benefit of the patients. His inclination toward the "thoughtful contemplation of what is seen" (Humboldt 1845: p.5, "*denkende Betrachtung des Beobachteten*"), nurtured by Müller, motivated his pursuit of observational natural science and zoology. He made notable contributions to this field, such as his research on opalines, tritons, urodeles, and the *Diplozoon paradoxum,* as well as on the "alveolar colloid of the liver" (in his doctoral dissertation) (see Klunzinger 1903, for a list of publications). In 1900, in recognition of his achievements, he was made an honorary member of the Leopoldina-Carolinian Academy.

Ernst v. Zeller is an important figure among the physicians and scientists who were mentored by Johannes Müller in his circle of followers ("Müller's Lab": Otis 2007). Although initially limited to the Württemberg area, Zeller's contributions to the medical and scientific fields are remarkable. The influence of Johannes Müller on the work of the Zeller family is evident in several ways. The transcript of a lecture on Comparative Anatomy given in 1851 provides tangible evidence of this influence.

## The lecture notes from 1851

To provide a comprehensive account of Johannes Müller's complex lecture on Comparative Anatomy, a detailed history of the subject in the 18th and early nineteenth centuries would require several years of work. This would need individual historical studies and studies of the development of the respective epistemological assumptions and structural findings up to the present day (Russell 1916; Jahn 2000; Hoßfeld et al 2003; Levit and Meister 2006), which would require the expertise of several specialists. In the following, a few illustrative examples are highlighted to demonstrate the potential of the lecture notes for future studies.

Johannes Müller took an extensive course load in Berlin, devoting four hours each week during the summer term to Comparative Anatomy, three hours to pathological anatomy, and six hours to human physiology (see Müller 1930a, b). During the winter semester, he devoted six to nine hours to human anatomy, and in addition to working on dissection exercises with the surgeon Friedrich Schlemm (1795–1859), he also devoted three hours to pathological anatomy (Koller 1958). A lecture on pathology given by Müller in Bonn in 1830 (Gágyor 2008) was recorded by Jakob Henle (1809–1885), who later became a professor of anatomy in Zürich, Heidelberg, and Göttingen and also published with Müller (1841).

In the summer semester of 1851, at the height of his scientific thinking and work, Johannes Müller gave a lecture on Comparative Anatomy, the transcript of which is discussed here. The transcript provides a comprehensive insight into Müller's perspective and is, therefore, an exceptional contemporary resource. At the end of the semester, Müller again traveled to Helgoland for a marine biology expedition, accompanied by the later famous morphologist Gegenbaur (1901). It is uncertain whether the student Ernst Zeller, who recorded the lecture, and Carl Gegenbaur had a personal meeting either in the Berlin "Theatrum Anatomicum" or in the "Anatomisch-Zootomisches Museum" of the university's main building. But the possibility remains.

In the second half of his life, Johannes Müller was known for his reluctance to discuss his philosophical views. This trait is reflected in his limited written or oral expression on the subject (Koller 1958; Otis 2007). However, his research findings were presented with great enthusiasm, as if he were trying to paint a complete picture of life by assembling its various components. The lecture lacked a proper introduction and conclusion and began abruptly with explanations (Folios 2r, 61r). Using a system of his own devising, the speaker gave the audience the impression of a comprehensive perspective. Nevertheless, it is worth noting that Johannes Müller's lecture on Comparative Anatomy was not a review of his research, but rather part of the body of knowledge available at the time. It is to say that Johannes Müller's academic teaching was honest and transparent, and he frequently referred to other authors. The transcript cites no less than 32 authors (Zeller and Werneburg 2024a: Table 2). It is worth noting that contemporary comparative animal anatomy still does not surpass the level of detail presented in Müller's lecture, with only evolutionary and embryological derivations being added today.

**Table 2:** Overview of the contents of the Lecture notes

Folio-page	German	English translation
2r	**Knochen der Eingeweide**	**Bones of the viscera**
2r	Zähne	Teeth
2v	Geweihe und Hörner	Antlers and horns
5r	**Scelet der Wirbelthiere**	**Vertebrate skeleton**
5r	Rückgrat. Wirbelsäule	Backbone. Vertebral column
6v	Schädel	Skull
8r	Schläfengegend	Temporal area
9r	Kiefergegend	Jaw region
11r	Sinnenzone	Sensory area
11v	Jochtheile des Kopfes	Zygomatic parts of the head
12v	Zungenbein und Kiemenapparat	Hyoid bone and gill apparatus
14r	Wirbelsäule	Vertebral column
15r	Rumpf	Trunk
16r	Schultergürtel	Shoulder girdle
16v	Die Jochverbindungen der Schulter	The zygomatic connections of the shoulder
16v	Becken	Pelvis
17v	Extremitäten	Extremities
19v	**Gefässsystem**	**vascular system**
20v	Gefässsystem der Polypen	Vascular system of polyps
20v	[Gefässsystem] bei den Medusen	[Vascular system] in the medusae
21r	[Gefässsystem] der Echinodermen	[Vascular system] of the echinoderms
22r	[Gefässsystem] der Würmer	[Vascular system] of the worms
23r	[Gefässsystem] der Articulata	[Vascular system] of the Articulata
23v	[Gefässsystem] der Mollusken	[Vascular system] of molluscs
24r	[Gefässsystem] der Wirbelthiere	[Vascular system] of vertebrates
24r	[Gefässsystem der] Fische	[Vascular system of] fish
25r	[Gefässsystem der] Amphibien	[Vascular system of] amphibians
25v	Venen	veins
26r	[Vena cava inferior], Vena umbilicalis	[Inferior vena cava], umbilical vein
26v	Akzessorische Herzen	Accessory hearts
27r	Wunderneze, Wunderneze der Carotis	Rete mirabile, Rete mirabile of the carotid artery
28r	Schwimmblase mit ihrem Wundernez	Swim bladder with its rete mirabile
29r	Lymphgefäße	Lymphatic vessels
30r	**Nervensystem**	**Nervous system**
31r	Würmer	Worms
31v	Mollusken	Mollusks
32r	Bivalven	Bivalves
32r	Cephalopoden	Cephalopods
32v	Articulata	Articulata
33r	Wirbelthiere	Vertebrates
33r	Fische	Fish
34v	Amphibien	Amphibians
34v	Vögel	Birds
35r	Säugethiere	Mammals
35v	Neurologie der Peripherischen Nerven	Neurology of the peripheral nerves
37r	Augenmuskelnerven	Eye muscle nerves
37r	Nervus sympathicus	Nervus sympathetic
38r	Sinnesorgane	Sensory organs
38v	Gehörorgan der Cephalopoden	Auditory organ of cephalopods
39r	Gehörorgan der Wirbelthiere	Auditory organ of vertebrates
40v	Gesichtsorgan	Visual organ
41v	Auge der Wirbelthiere	Eye of vertebrates
43v	Geruchsorgan	Olfactory organ
44r	Geschmackswerkzeuge	Taste sensors
44v	**Splanchnologie**	**Splanchnology**
44v	Athemwerkzeuge	Breathing tools
46r	Wirbelthiere	Vertebrates
47r	In der Luft lebende Thiere	Animals that live in the air
48v	Stimmorgan	Vocal organ
50r	Verdauungsorgane	Digestive organs
52r	Wirbelthiere	Vertebrates
53v	Drüsen des Verdauungssystems	Glands of the digestive system
54v	**Harnwerkzeuge**	**Urinary tools**
55r	**Generationsorgane**	**Sexual organs**
59r	Geschlechtsorgane der Wirbelthiere	Sexual organs of vertebrates
60v	Penis	Penis
61v	**Ausscheidende Drüsen**	**Excretory glands**

The main headings are highlighted. r = recto (front), v = verso (back). German spelling as in the original

Emil du Bois-Reymond (1858: p. 272), one of the great scholars from the Müller laboratory (Finkelstein 2013; Otis 2007), described Müller's lecturing style as follows: "He consistently presented concise content that was drawn from comprehensive knowledge. Müller never lost his train of thought, repeated himself, or made a slip of the tongue. Despite scanning the room with his piercing eyes, which even fixed on him as an unwelcome intruder, his speech was delivered calmly, clearly, and plainly. The message was straightforward and easily understood, so much so that it could have been transcribed verbatim and given straight to the publisher."^1^ He had honed his skills in his lectures on Comparative Anatomy and was also proficient at illustrating on the blackboard.

Ernst Zeller's transcript was written in personal shorthand, with many words abbreviated. The transcript closely reflects the original wording of the lecture. Ernst Zeller was also a skilled draftsman, as shown in his later works (cited by Klunzinger 1903, and by Zeller and Werneburg 2024b), so the drawings of the lecture are likely an accurate representation of the original.

The lecture was divided into the following topics: Visceral Bones, Vertebrate Skeleton, Vascular System, Nervous System and Sensory Organs, Splanchnology (Respiratory and Digestive Organs), Urinary Organs, Generative Organs, and Excretory Glands (Table 2). The "typical" basic characteristics of the forms, along with their functionality, were characterized by discussing the individual chapters in all "animal" groups, from man to the “Infusoria”, according to Müller's system.

The classification system for animals followed the understanding of the time, with "Articulata" (articulated invertebrates) typically being grouped together because of their similar morphological features. Vertebrates were primarily represented by "*Amphioxus*," known as the lancelet, as the "archetypal" example. Fish and terrestrial vertebrates were then systematically arranged according to their level of complexity within the *Scala naturae* (e.g., Folio 5r). Müller identified groups with unique characteristics, rather than placing them in strict hierarchical order from "lowest" to "highest" or vice versa. At times, he organized groups based on the level of organization of the organs rather than taxonomic categorization. In the nineteenth century, "amphibians" were typically divided into "unscaled" and "scaled" groups (Hoffmann 1890). The former group included true amphibians such as caecilians, frogs, and salamanders and their relatives (Caudata), while the latter group consisted of reptiles such as turtles, lizards, snakes, and crocodiles as they are currently understood (e.g., Folio 12r). Birds were thought to represent the transition to the mammalian "stage" (e.g., Folio 13v). It was not until later that Huxley (1864) showed, from a Darwinian perspective, which birds evolved from reptilian ancestors (Modesto and Anderson 2004).

In the context of the *Scala naturae*, man was once considered the last rung. The fascinating tension between the *Scala naturae* and the prevailing concepts of the 1850s is exemplified by certain statements such as "with amphibians it remains so, while with mammals it is already different…" (e.g., Folios 15v, 17r, 17v, 18v, 20r, 25v). Since the Cuvier-Geoffroy debate of 1830, evolutionism has been a controversial topic. Did Müller contradict his principles in this case? The developmental stages of the lower levels of the *Scala naturae* also serve as an explanatory model for Müller during ontogeny. As an illustration, he noted the similarity between the brain of amphibians and the fetal brain of humans (Folio 34v). It should be noted that embryology only briefly touched on organ development (fetal development) and was not the focus of the lecture. Müller ignored early developmental stages such as cleavage and gastrulation (v. Baer 1828/37) because of their limited importance for functional morphology (but see Folio 3r).

In the first chapter, "Visceral Bones" (Folio 2r), Müller examined teeth, antlers, and horns (composed of keratin), as well as the functional aspect of bone pneumatization (calcium phosphate) in vertebrates. In addition, the study includes all identified calcium carbonate shells and exoskeletons of invertebrates, such as the cuttlebones of cuttlefish (Cephalopoda; "Os sepiae"; Folio 3v) and the carbonate and silicate shells of unicellular infusorians (Protozoa). Müller's mention of the chemical composition of biominerals is consistent with the understanding of his time (Folio 2r; Schmidt 1849).

In the chapter "Skeleton of Vertebrates," Müller followed the Goethe-Oken vertebra theory for the skull. Based on his studies of the hagfish, he proposed a structure consisting of three vertebrae and intermediate parts (Folio 6v). In particular, Müller recognized the research of his former student and successor in anatomy, Karl Bogislaus Reichert (1811–1883), on embryonic modifications of the gill arches (visceral arches) (Reichert 1838; Gaupp 1913; Zeller 1989, 1993). He originally praised it in "Müller's Archiv" (1838: quoted in Russell 1916) and in the "Handbuch der Physiologie" (Müller 1833ff [1837], vol. 2: p. 738). However, it was not considered in his lecture. Instead, Müller informed the audience that the auditory ossicle (columella) of "scaled amphibians" (reptiles) and birds corresponded to the three auditory ossicles found in mammals (Folio 40r; Fig. 3E, F), ignoring Reichert's view that only the stapes corresponded to the columella in mammals (Gaupp 1913). It is possible that the results were too recent to be passed on as common knowledge. Müller also rejected the distinction between dermal and replacement bones (Schmidt 1849; Kölliker 1861), as well as the division of the skull into neuro- and viscerocranium (C.G. Carus 1807, 1828: quoted after Russell 1916). Instead, he considered the visceral arches, such as the jaw and hyoid, to be "legs of the head" (Folio 8r). According to him, they were appendages of the vertebral structures that supposedly extended into the skull.

**Fig. 9:**
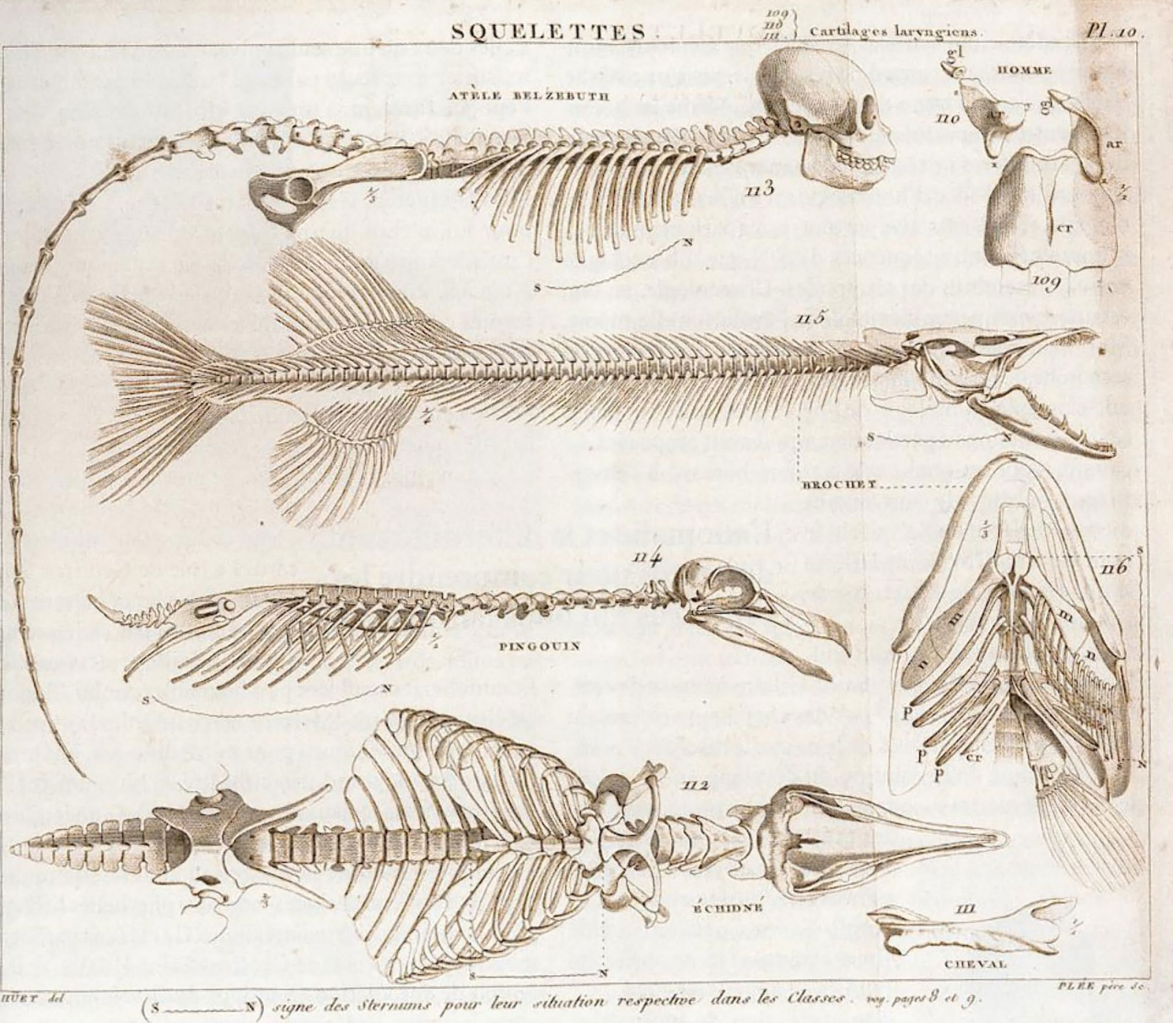
Comparison of the skeletal “Baupläne” of the golden-fronted spider monkey (No. 113; *Ateles belzebuth*), of the pike (No. 115–116; *Esox lucius*), a penguin (No. 114), and an echidna (No. 112). From: Geofroy Saint-Hillaire (1818)

In a few cases (Folio 30r), to reveal and clarify form-function relationships, Müller made comparisons between conditions of different "types" that we now know are genealogically distant and thus not systematically closely related. One comparison was between vertebrates and arthropods. Müller explained that vertebrates originally had six limbs, two of which were the jaws (Fig. 5C). "The leg of the head sits on the squamosum," according to the lecture notes (see Folio 8r; Fig. 4A). This analysis follows the holistic approach of Geoffroy Saint-Hilaire (Fig. 9) and is in line with the "construction plan" principle as exemplified by Goethe. In particular, recent studies on *Hox*-genes have given new meaning to what was previously considered a nonsensical homologization of body segments (e.g., Papageorgiou 2007). *Hox*-genes found in vertebrates and arthropods regulate the shape and position of body appendages or the longitudinal structure of the body axis. In addition, Gegenbaur (1864/65) developed the gill arch theory of the origin of pectoral fins, which is relevant in this context (see Brazeau et al. 2023). E. Zeller noted from the lecture that the needlefish *Belone* has several "legs," which would correspond to the pectoral rays, further supporting the present discussion (Folio 17v). Here, as with the vertebral theory of the skull, Karl Friedrich Kielmeyer's (1765–1844) principle of repetition of parts certainly applies (Russell 1916; Schumacher 1979).

Müller also compared vertebrates to arthropods when discussing the nervous system. He discussed the arthropod abdominal cord as a homolog of the vertebrate spinal cord (Folios 30r, v; see Fig. 3E) and mentioned a "cauda equina" (Folio 33r), a typical mammalian collection of spinal nerve roots located at the end of the spinal cord.

The section on the "Peripheral Cranial Nervous System" (Folio 35v) may prove challenging due to the unclear and confusing interpretations presented in the margin notes of the student's college book (Fig. 3E). It is evident that both the lecturer and the listener found this subject difficult to understand, which is not unexpected. Dietrich Starck (1908–2001) clarified the homology of peripheral (head) nerves (Starck 1979) by emphasizing the importance of considering both the central origin and the peripheral route, as well as the area of innervation. The peripheral route can be influenced by various topographical anatomical factors and spatial conditions of individual nerve tracts. Nerve tracts may sometimes converge without any homology.

Müller attempted to explain the homology (as per Owen!) of cranial and spinal nerves by adhering to the vertebral theory of the skull (Folio 35v; Figs. 4B, D; 5A, B) (see also discussion in Richards 2018, 2020). This explanation paved the way for the segmental head theory of Carl Gegenbaur and Max Fürbringer (1846–1920) (Goodrich 1930; Veit 1947; Starck 1979; Mitgutsch 2003). Important findings in this field were discovered long after Müller's research, including the importance of the neural crest and placodes in the development of the cranial nervous system (Starck 1982; Gans and Northcutt 1983; Hall 2009; Gilbert 2013).

At this point, Müller offered a constructive yet critical perspective on the experiment. The discussion revolves around experiments conducted on bird brains in which injury to the semicircular canals induced rotational movements. Müller emphasized that in such experiments, the flocculus cerebelli is damaged, and this alone leads to rotational movements (Folio 35r).

Naturally, Johannes Müller prioritized topics from his research, such as ganoid fish, bird, and echinoderm anatomy. These included the microscopic structure of the notochord (chorda dorsalis), which reaches the skull in the hagfish (Myxinoidea) (Folio 5r); the structure of the vertebral column together with the "metamorphoses" of the ribs (Processus costales et accessorii) (Folio 6r); the extremities (Folio 17v) and branchial arches (Folio 46r); the vascular system of echinoderms (Folio 21r) and flatworms (Folio 22r), and the accessory hearts of annelids (Folio 22v); and the blood nets (rete mirabile) in the wall of the swim bladder of fish (Folio 28r). The life cycle of jellyfish, including the developmental changes between polyps and medusae (Folio 55v ff), and the functionality of their cnidocytes (Folio 50v) were discussed in detail. Müller emphasized the importance of organs such as the auditory sacs and pigment cup ocelli in the sensory system (Folio 38r ff), the vocal organ in the respiratory system, and, famously, the Müllerian ducts (Folio 59v) in the reproductive system (see Fig. 3C, D). Finally, the lecturer concluded with a comprehensive explanation of various glands, with special attention to the skin glands (Folios 61r, v).

**Fig. 10:**
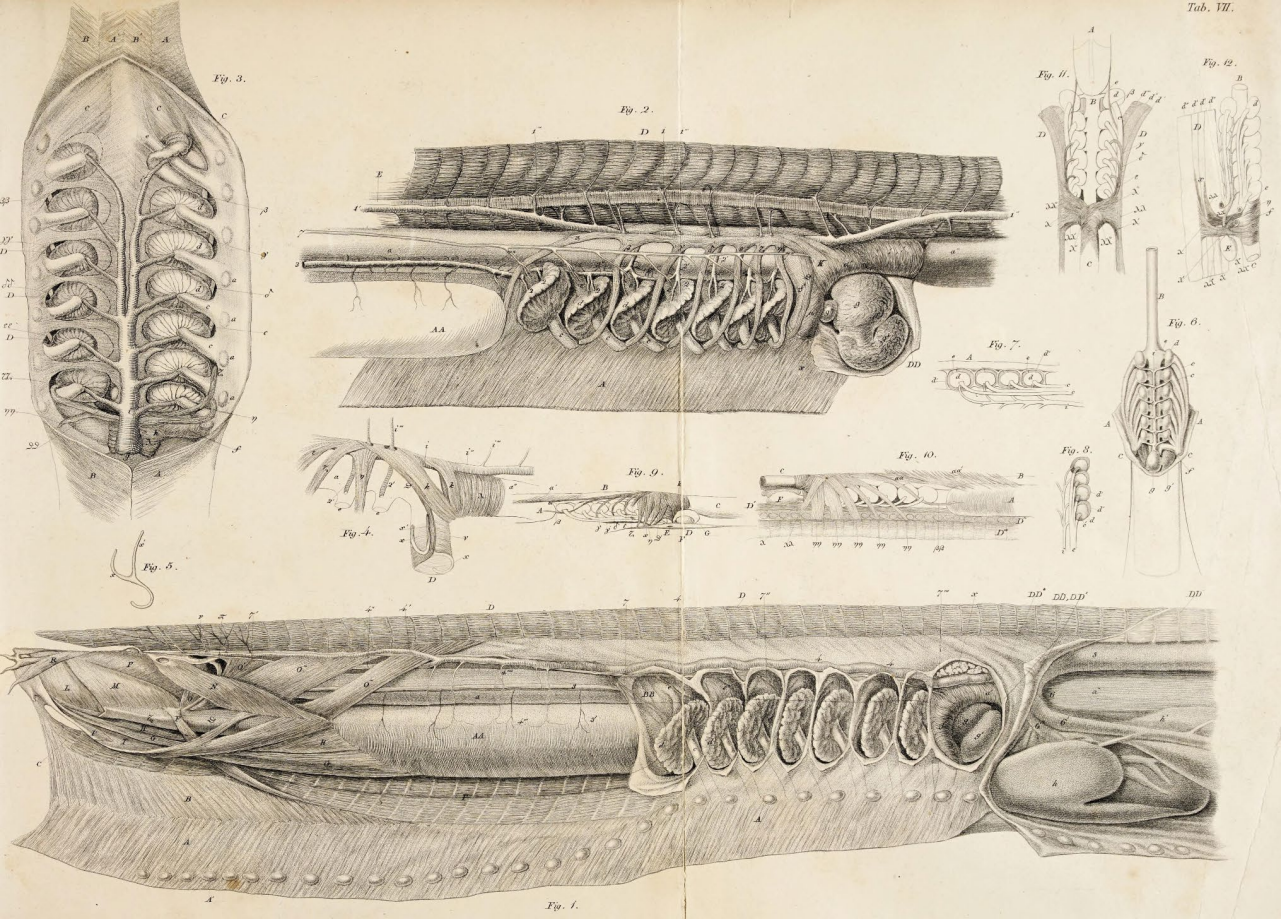
Lithographs from Müller's works. “On the Comparative Anatomy of the myxinoids” (mucus eels) (Müller 1834: plate VII of the 1st part) various views of the gill apparatus of the sixgill murre eel *Eptatretus (“Bdellostoma”) heterotrema*

It may seem surprising that the lecture did not include a section on myology, the study of muscles. However, this may have been due to time constraints. It is worth noting that the author treated this topic extensively and competently in other works, such as the monograph on the Myxinoidea (Müller 1834; see Fig. 10) and the "Handbuch der menschlichen Physiologie" (1833ff; Fig. 6).

All in all, the lecture notes on comparative zoology provide an excellent insight into the comprehensive knowledge of this subject in the middle of the nineteenth century. Johannes Müller continued to argue in accordance with Aristotle's *Scala naturae*, but did not slavishly adhere to it. He arranged the taxa according to the increasing complexity and functionality of the organs. Embryology was not a primary focus, as it does not contribute significantly to the functional anatomy of the adult. Müller adhered to the vertebral theory of the skull proposed by Goethe and Oken, which was the most contemporary, i.e., typological, understanding at the time. It is noteworthy that Müller disseminated much of his research in his lectures, not out of self-interest, but because he had made a significant contribution to the field of zoological knowledge.

## Epistemology of Johannes Müller

### Position on the idea of evolution

It is difficult to place Johannes Müller's position within the late Enlightenment and the Goethean era of the eighteenth and nineteenth centuries. This is due to the various approaches he adopted throughout his life, ranging from the romantic (Müller 1826b) to the strictly empirical (Müller 1848) and more precise (Haberling 1924; Koller 1958). Johannes Müller had the remarkable quality of remaining impartial to contemporary intellectual movements, including the burgeoning theory of evolution. During the famous Cuvier-Geoffroy debate of 1830 between George Cuvier (1769–1832) and Étienne Geoffroy Saint-Hilaire (1772–1844) (May 1919), he took a mediating position, as documented by Du Bois-Reymond (1858) and J.V. Carus (1872: p. 636). Guided by his concept of "idealistic vitalism" (see below), the author relied primarily on his precise observation of nature, coupled with an aptitude for accurate documentation. His comprehensive research approach surpassed the typology of his time (Levit and Meister 2006), enabling him to gain profound insights into the fundamental laws of life, further enriched by his study of living organisms as part of his marine biology research. Thus, fueled by his immense passion for his field of research, and after conducting 19 research expeditions, including trips to Helgoland and the Mediterranean as examples, Müller and his students successfully created a comprehensive worldview (Otis 2007). The remarkable aspect of their work lay in the substantial amount of data presented in a coherent and commanding manner that captivated both the audience and the reader.

Johannes Müller sought to understand the diversity of life in its entirety, deliberately excluding inanimate nature. He also studied the essential characteristics of animals in terms of their structure and function. During his early years, he adhered to the principles of the Romantic *Naturphilosophie* that was prevalent in the late 18th and early 19th centuries. He maintained his Roman Catholic faith and attended the Gymnasium in Koblenz, Germany, where he was a student of the *Naturphilosoph* and Catholic writer Joseph Görres (1776–1848). With reference to Aristotle (384–322 B.C.), he also supported vitalism (Meyer-Abich 1949), which holds that a life force flows through all organisms.

Müller certainly noted the emergence of the concept of evolution, although Lamarck probably came closest to his views (Russell 1916). However, Johannes Müller did not incorporate the concept of evolution into his research and worldview. One possible explanation, apart from his fundamental religious beliefs, is the speculative nature of evolutionary research, which ran counter to his empirical claims. Müller's scientific approach did not include elements such as "transformation" or "speciation," except for the integration of evolution and his connection to the Church's doctrine of creation. He believed that there were no transitions between species, but to account for changes over time and space, he had to uphold the ideals of *Naturphilosophie* regarding "type" and "metamorphosis." The concept of polarity was not applicable in the context of temporalization (Lepenies 1976); instead, he adhered to Aristotle's *Scala naturae *spatial reference system (Rapp 2013ff). With Geoffroy (Mayr 1982; Hallgrímsson & Hall 2011), Müller considered the state of an organ approaching type or ideal form as its highest level of development. He considered the human brain to be the most advanced and that of other mammals to be "reduced" (Folio 33r).

### Homology and type

Johannes Müller probably used the terms "homology" and "analogy" with reference to the "doctrine of similarity" as formulated by Sir Richard Owen (1804–1892) (see Rupke 2009). Owen (1843) defined structures with similar placement in the body as homologous (see Nowikoff 1935; Starck 1950; Hoßfeld and Juncker 1999; Hoßfeld and Olsson 2003; Werneburg & Hoßfeld 2024). The discovery of anatomical similarities in nature has a long history, as illustrated by the famous skeleton comparisons of Belon (1555) or Geoffroy Saint-Hilaire (1818) (Fig. 9). The basis for this understanding goes back to Plato (428/427–348/347 B.C.), who postulated that the similarities found in nature stem from a transcendent principle, the world of ideas. This view, with modifications, also entered Christian dogmatics (Albert 2008). Consequently, organisms possess similar "blueprints" that derive from this principle. In the early days of Comparative Anatomy, Georges Cuvier, Johann Wolfgang von Goethe, and Richard Owen were important influences. This view was permeated by German idealism, which found new philosophical support. In a conversation with the poet and historian Friedrich Schiller (1759–1805) on July 20, 1794, in Jena, Germany, Goethe admitted to taking the ideas literally when comparing similar organisms (Goethe 1982: p. 112). Müller relied solely on empiricism to discover the "type" and, mirroring the approach of evolutionists, distanced himself from Goethe's speculative method of investigating nature through observation and intention. Moreover, the personal relationship between Goethe and Müller was relatively reserved (Koller 1958).

At the beginning of the nineteenth century, German Romanticism underwent a transformation that led to an emotional elevation of the concept of nature—as seen in the works of Novalis (1772–1801) and Carl Gustav Carus (1789–1869)—that moved away from strict natural science. Johannes Müller, like many of his contemporaries, was confronted with this situation and dedicated his life to the holistic understanding of living nature. Müller's mission was to understand living nature as a complete and integrated system.

With Charles Darwin's (1809–1882) theory of evolution, the concept of homology received a new methodological basis (see, for example, Hoßfeld and Olsson 2005). According to this concept, similar organs can only be considered homologous and structurally identical if the organisms in question share a common ancestry (Darwin 1859). For example, the legs of insects and mammals share the basic function of locomotion in Müller's sense. However, from a phylogenetic perspective, these organs arose independently and in different lineages. The development of the scientifically comprehensible and justified method used today is rooted in the evolutionary thinking of many of Johannes Müller's contemporaries, even before the time of Darwin. This thinking was further inspired by the work of the influential anatomist Carl Gegenbaur (Gegenbaur 1878; Hoßfeld et al 2003). Hennig (1950), Remane (1952), and Wagner (2014), among many others, have since refined and extended this approach. Due to the historicization of nature, both paleontological and ontogenetic aspects have been incorporated into the interpretation of evolutionary processes. This was demonstrated by Ernst Haeckel (1834–1919), as recently discussed by Levit et al. (2022) and Werneburg et al. (2022a, b, c).

Johannes Müller did not adopt a structure- and feature-based interpretation of the concept of homology, as used in evolutionary research. First, Müller grew up in a Catholic tradition that recognized the omnipresence of created nature. Müller, who was undoubtedly familiar with the embryological research of Carl Ernst von Baer (1792–1872), considered ontogenetic processes to be merely cyclical. Fossils known to the author through the work of Cuvier (e.g., Cuvier 1812) and his investigations (Müller 1846, 1849) were either perceived as natural defaults or taken as evidence of the antediluvian world mentioned in the Old Testament, but not as evidence of an evolutionary narrative.

Müller resisted all attempts to explain nature as a product of deep-time natural processes (Gould 1987) because of his sociocultural embeddedness in the history of the Western world. From a contemporary perspective, one could argue that Johannes Müller is a tragic figure in the History of Science. He did not have the same “luck” as Haeckel in being exposed to the theory of evolution and was hindered by traditional beliefs that prevented him from uncovering the desired understanding of all living nature. It is symbolic that Müller died only one year before the publication of Darwin's "Origin of Species" (Darwin 1859). He was born after the time of Goethe, but was not influenced by Goethean epistemology (Breidbach et al. 2001). However, Müller's worldview represents a self-contained explanatory framework in which the "world soul," the Christian faith, and the intellectual-historical trends of his era defined the limits and boundaries within which this exceptional natural scientist could develop, as if in a test tube, a self-contained system. This approach was based on a comprehensive understanding of living organisms. Anatomical evaluation is crucial, but equally important is the evaluation of functionality and integration into the environment—a very contemporary view (Uexküll and Kriszat 1962).

### From experiment to pure insight

Throughout his life, Johannes Müller consistently improved and refined his research approach. He gradually moved away from speculations of *Naturphilosophie*, as shown in his dissertation on the laws of motion in the animal kingdom (Müller 1822), and eventually avoided them altogether. His encounter with the rationalist Carl Asmund Rudolphi (1771–1832) in Berlin between 1823 and 1824 was particularly influential (Table 1; Koller 1958). In addition to the achievements of his students and colleagues (e.g., Rudolphi, v. Helmholtz, Henle, Schleiden, Schwann, Virchow), the work of Johannes Müller laid the foundation for modern medicine, allowing it to break out of its speculative environment into a more scientifically oriented field (Otis 2007).

Already in his inaugural lecture in Bonn, he rejected Romantic *Naturphilosophie* and stuck to the theory of types (Müller 1824). For him, however, only experiment and observation led to type and ideal, never speculation or intuition. Here, he distanced himself from his contemporary Carus (1822), who had chosen speculative observation of nature as a heuristic principle, although he also liked to work abstractly with geometric figures and the ideal in phenomena outside of living nature (e.g., magnetism, electricity). For Müller, the only methodological tools were exact observation of natural phenomena and experiments, although he had developed a very critical relationship to the latter and set high standards since any experimental intervention also carries the risk of violating the natural phenomena of life and falsifying the answer (see below; Müller 1824). The increasing rejection of experiments probably has another origin. While the natural scientist Goethe largely rejected measuring instruments because they contradicted the natural cognitive sense of the human eye, Müller rejected experiments because they led to atomization and thus to the destruction of life processes. The experiment was also symptomatic of the fragmentation of holistic physiology into its analytical subdisciplines, which, unlike him, lost sight of the big picture.

Müller often and readily referred to Cuvier's traditional approach, including in his own letter of recommendation to Berlin in 1833 (Du Bois-Reymond 1858; Koller 1958: pp. 99–102). He followed Cuvier not only in the general theory of construction, but also in the law of correlation between similar groups of organisms and the catastrophe theory of multiple extinctions in history (see Folio 16r). He considered functional new formations of structures quite possible, such as the cartilages of the mouth region of hagfish (myxinoids; Müller 1834). However, Müller rejected the more static morphology of Geoffroy Saint-Hilaire (1818–1822; Fig. 9), which was based on "construction plans," as well as the purely mechanistic ("atomistic") modern physiology, which is based solely on experience with insufficient consideration of function.

However, Müller was not a pure "Cuvierian." He also recognized the Goethe-Oken vertebral theory of the skull (Peyer 1950) in his hagfish monograph (Müller 1834). He also used the embryological criterion of von Baer (1828/1837), according to which the "archetype" often appears most clearly in early embryogenesis (Russell 1916). He was also able to demonstrate the "homology" of the notochord (chorda dorsalis) in all vertebrates and the lancelet fish ("*Amphioxus*") (Folio 5r ff; see Fig. 3B). He had also discovered animal cells in this organ and, based on cell theory (Schleiden, Schwann, Virchow), considered cell tissue to be a fundamental characteristic of life. In plants, he argued, this tissue is characterized by closed cells; in animals, except for the adipose tissue, the vitreous body, and the notochord, it is syncytial. At this point, Müller saw the danger of reductionism, of an "atomistic" view of nature, which would later be taken up by his student Rudolf Virchow and his "cellular pathology." The holistic picture of the organism was rapidly lost and dismembered, and it was the particular achievement of Carl Gegenbaur to counterbalance this with comparative and, increasingly, evolutionary morphology (Hoßfeld et al. 2003; Maier 2021).

### The search for the “original phenomena”: confusion, doubt, and mystery

In Müller's worldview, evolutionary theories were considered to belong to the realm of purely hypothetical and unprincipled inquiry. Müller followed a path of calm and dispassionate observation, using the comparative method and basic experiments that underwent numerous modifications to arrive at the "primordial phenomena" of life (Müller 1824: p. 272). These phenomena possess a divine quality that can only be perceived by scientists through a combination of observation, experimentation, and a philosophical-idealistic approach. Their beauty, especially in their morphology, is as striking as their functionality. Johannes Müller's study of nature therefore had an almost religious quality. According to Müller, the physiologist has two ways of seeing: an observing eye and a spiritual eye. Philosophy comes into play only in relation to empirical sensory knowledge, but does not dominate it. The observing eye provides the most precise description of the phenomena, while the spiritual eye—guided by the phenomena—delves deeper into the observation (Müller 1833ff: Prolegomena). In this way, Müller described the basic characteristics of life, such as growth, development, irritability, reproduction, respiration, and cell tissue, on the way to an exact physiology. For animals, there were additional attributes such as sensation, movement, and digestion. These qualities (*Vis vitalis*) emphasize the life force's role as a creative force that intentionally transforms matter. It was Johannes Müller's belief that medicine could only begin with "correct physiology," and his responsibility was to combine appropriate philosophical and physiological training with the practical skills of the physician and natural scientist (Müller 1824).

Lecture notes and academic literature indicate that Johannes Müller experienced "confusion, doubt, and mystery" with a purely anatomical approach (Otis 2007). As Müller continued to study the anatomy of organisms, he became increasingly aware of their structural differences. Although evolutionary homology research could have clarified these differences, it would not have provided a comprehensive understanding of nature as Müller defined it. He believed that the key to understanding life lay in the observation of nature, and he devoted his life to pursuing this approach. He rejected the speculative nature of evolutionary thinking, as well as that of the natural romanticism mentioned earlier. In Müller's worldview, understanding could not be reconstructed through thought alone; it had to be discovered through observation, without resorting to Goethe's intuitive speculation. In this context, Müller argued that the concept of the world soul in his worldview should not be seen as speculative, but rather as an observation that all organisms are imbued with life. Müller was convinced of this, and his search for truth was based solely on his research. "Confusion, doubt, and mystery" were not expressions of doubt or lack of understanding of a superior model of nature, but rather an intrinsic, virtuous, and investigative motivation to delve deeper into the mysteries inherent in life. In this respect, the research approach of Johannes Müller can serve as an example. Without advocating its adoption, his method illustrates the contribution that a self-contained worldview can make to achieve the highest levels of scientific and motivational excellence. This can also be observed in Friedrich von Huene (1875–1969), a deeply religious paleontologist whose research motivation was to understand and depict God's magnificent creation (see Werneburg 2021).

The diversity of living organisms should be ordered according to a principle or an idea. Since Müller focused on living things, organ function was crucial to him. Therefore, he structured his lecture to discuss all known forms in the animal kingdom, including the central nervous system (see Folio 30r) and the visual system (Folio 38r). Although homology includes structure, it was only one aspect of Müller's lecture, as outlined in the lecture notes. The role of the organ's functionality in life was important to Johannes Müller. While his work showed his confusion and doubt regarding intrinsic order, which presented several puzzles (Otis 2007), Müller's lecture notes demonstrate his effective influence on his students. He provided a clear structure and confidently prepared inexperienced physicians and budding scientists for their journeys of continuing inquiry. Nevertheless, there remains an uncertainty about the accuracy of this presentation, a doubt about one's ability to comprehend, that continues to shape the post-Darwinian era and actively encourages further research.

Johannes Müller's overarching worldview was short-lived as the sciences became increasingly specialized. After his death, his chair and its associated disciplines were divided into anatomy (Reichert), physiology (Helmholtz, du Bois Raymond: electrophysiology), pathology (Virchow: cytopathology), cell biology (Schwann: histology), and zoology (Haeckel: evolutionary biology). This division facilitated the development of reductionistic and "atomistic" research. Johannes Müller's zoological-zootomical collection was not preserved in its entirety, as it was divided into two sections. The first section was a zoological-systematic collection that was housed in the newly founded Natural History Museum in Berlin in 1889. The second section, an anatomical-zootomical collection, was housed in the anatomical department of the university (Otis 2007). This division led to the destruction of the unified collection that Müller had amassed. His students took no action to preserve his universal view of the world. However, they respectfully expressed their admiration for him (Otis 2007), fully aware that their progress was rooted in the teachings of their tutor (Du Bois-Reymond 1858). This foundation of knowledge continues to serve as an essential element of personal development in various fields today.

Amidst the increasing fragmentation of molecular biological research in recent years, the appeal for holistic thinking in biologically oriented sciences has increased (Ziemke 2015; Zeller et al. 2019; Maier 2021). A thorough examination of Johannes Müller's life and work can provide valuable insights and inspiration. His remarkable ability to carefully observe nature and his reverence for all living organisms will continue to serve as our guiding principles and benchmarks.

## Conclusions

1. Johannes Müller's lecture notes on comparative zoology provide a unique insight into the knowledge of the subject in the mid-nineteenth century. They are a valuable source for the history of various morphological topics, including the anatomy of the skeletal, vascular, and nervous systems. Of particular note are the treatment of bones in relation to the vertebrate and the vertebral archetype, and the discussions of Müller's discoveries, including echinoderm and fish anatomy, the rete mirabile, and "Müller's ducts."
2. In addition to authorized published references, a more complete picture of the great "physiologist" (i.e., life scientist, biologist in modern terminology) Müller can be drawn regarding his personal philosophy and scientific approach. As such, Johannes Müller can be seen as a researcher between the times of the Romantic *Naturphilosophie* and the Darwinian Revolution. He did not integrate both approaches but followed his own.
3. After being influenced by Joseph Görres and other Romantic *Naturphilosophen* in his earliest works, he distanced himself from all speculative approaches to science, including the early pre-Darwinian attempts to understand nature from an evolutionary point of view. Müller had a vitalistic understanding of nature going back to Aristotle and he followed an idealistic interpretation of natural beings (Plato, Goethe, Schelling, Oken). This approach was also influenced by his Catholic faith.
4. Müller pursued a rigorous empirical research agenda, striving to understand life in its entirety and nature as a whole. To this end, he used the methods of homology and analogy to distinguish and identify similarities in the structures of organisms. Furthermore, his rationalism required a rigorous skepticism of experimental approaches, since any treatment would carry the highest risk of affecting the functionality of an organism by altering natural conditions.

## Data Availability

No datasets were generated or analyzed during the current study.

## Footnotes

1 Translated by us from: “Er war sich stets gleich an gedrungenem, aus vollkommener Sachkenntnis zweckmäßig geschöpftem Gehalt. Müller verirrte, wiederholte, versprach sich nie. Während sein durchdringendes Auge durch die Versammlung schweifte, auch wohl einem Eindringling zur Pein auf ihm ruhte, floß aus seinem Mund die Rede ruhig, klar, schmucklos gediegen, so daß sie, stenographiert, ohne weiteres hätte in die Druckerei wandern können.“
